# Development of Real-Time Water-Level Monitoring System for Agriculture

**DOI:** 10.3390/s25175564

**Published:** 2025-09-06

**Authors:** Gaukhar Borankulova, Gabit Altybayev, Aigul Tungatarova, Bakhyt Yeraliyeva, Saltanat Dulatbayeva, Aslanbek Murzakhmetov, Samat Bekbolatov

**Affiliations:** 1 Department of Information Systems, M.Kh. Dulaty Taraz University, Taraz 080000, Kazakhstan; gs.borankulova@dulaty.kz (G.B.); bsh.eralieva@dulaty.kz (B.Y.); ssh.dulatbayeva@dulaty.kz (S.D.); sb.bekbolatov@dulaty.kz (S.B.); 2 Department of Radio Engineering, Electronics and Telecommunications, International Information Technologies University, Almaty 050040, Kazakhstan; g.altybayev@iitu.edu.kz; 3 School of Information Sciences, University of Illinois Urbana-Champaign, Urbana, IL 61820, USA

**Keywords:** water resource management, velocity measurement, low-cost sensors, water level, internet of things

## Abstract

Water resource management is critical for sustainable agriculture, especially in regions like Kazakhstan that face significant water scarcity challenges. This paper presents the development of a real-time water-level monitoring system designed to optimize water use in agriculture. The system integrates IoT sensors and cloud technologies, and analyzes data on water levels, temperature, humidity, and other environmental parameters. The architecture comprises a data collection layer with solar-powered sensors, a network layer for data transmission, a storage and integration layer for data management, a data processing layer for analysis and forecasting, and a user interface for visualization and interaction. The system was tested at the Left Bypass Canal in Taraz, Kazakhstan, demonstrating its effectiveness in providing real-time data for informed decision-making. The results indicate that the system significantly improves water use efficiency, reduces non-productive losses, and supports sustainable agricultural practices.

## 1. Introduction

The limited availability of water resources and the growing need for the sustainable use of natural resources make effective management a key aspect of the agricultural sector. Modern agriculture faces a number of challenges related to the monitoring and control of water resources. The increasing demand for food, changing climate conditions, and the expanding shortage of water supplies necessitate the implementation of innovative approaches to ensure their rational use. Water resources play a crucial role in the development of the agro-industrial complex, supporting the sustainable growth of agricultural production. However, the inefficient management and irrational use of water lead to reduced crop yields, soil degradation, and increased water supply costs.

One of the most promising directions for addressing this issue is the application of modern digital technologies, particularly big data-processing technologies. The analysis of large volumes of data from various sources, such as meteorological services, soil moisture sensors, satellite imagery, and automated control systems, enables the acquisition of timely and accurate information for informed decision-making. Data-driven intelligent monitoring systems can provide precise irrigation planning, water consumption forecasting, and the identification of inefficient zones and water losses. Despite significant progress in the digitalization of agriculture, there remain unresolved challenges related to the integration of heterogeneous data, ensuring data accuracy and timely processing, and developing adaptive models for water resource management. In this context, the development of a model and monitoring system for effective water resource management based on big data processing technologies is a relevant task that could increase the productivity of the agricultural sector and reduce its environmental impact.

Kazakhstan is an agrarian country with significant natural resources and strong potential for agricultural development. Thanks to its vast fertile lands and diverse climatic zones, Kazakhstan is a major producer of grain crops, primarily wheat, as well as meat, dairy, and other agricultural products. The agricultural sector is a vital part of Kazakhstan’s economy, not only meeting the country’s domestic food needs but also positioning it as a significant player in the global agricultural market. The effective use of water resources plays a key role in the development of this sector. The issue of water scarcity in Kazakhstan is one of the country’s most critical environmental and economic challenges. Key factors affecting the state of water resources include limited natural reserves, the transboundary nature of water sources, increasing water consumption, and the impact of climate change. According to World Bank forecasts, Kazakhstan’s annual water resources are expected to decrease from 90 to 76 km^3^ by 2030 [1]. This means that within just eight years, the country could face a water deficit of approximately 12–15 km^3^ per year, around 15%. Among Central Asian countries, Kazakhstan is the most water-scarce due to its climatic conditions. In terms of water availability, Kazakhstan significantly lags behind Kyrgyzstan (245,000 m^3^/km^2^ per year), Western Siberia (198,000 m^3^/km^2^ per year), and even Turkmenistan, much of which is desert [2]. Approximately half of Kazakhstan’s water resources come from transboundary rivers whose sources lie outside the country.

Agriculture is one of the largest consumers of water resources in Kazakhstan. Effective water resource management in agriculture is critically important for ensuring food security, sustainable development, and ecosystem stability. According to [3], up to 90% of the annual runoff of steppe rivers occurs in spring, and up to 70% of mountain river runoff occurs in summer. The total volume of water resources in the republic is estimated at 100.5 km^3^, of which the available volume for economic use is 43 km^3^. The specific water availability in the country is 37,000 m^3^ per km^2^ and 6000 m^3^ per person per year. Kazakhstan is classified among Asian countries with insufficient moisture, and its central and southern regions fall into arid zones. As a result, the main area of agricultural land reclamation is irrigated land, which, when used properly, allows for stable crop yields regardless of natural conditions [3]. According to the land balance as of 1 November 2023, the Republic has 2.3 million hectares of irrigated land, of which 1.9 million hectares (82.3%) are agricultural lands, 206.3 thousand hectares (8.8%) are within settlements, and 186.8 thousand hectares (8.0%) are reserve lands [4].

In recent years, there has been a steady increase in average annual temperatures due to global and regional climate warming, which exacerbates the issue of efficient water use, especially in agriculture, leading to increased demand for water resources, including higher irrigation norms for crops [5]. A serious obstacle to solving the problem of rational use of water resources in our region is the current lack of fully functioning water resource monitoring systems that allow for the continuous collection of hydrological data, their analysis, and the subsequent formation of forecasts using big data technologies. The situation is aggravated by the fact that the collection and processing of hydrological information is carried out using physically obsolete equipment, which leads to distortions and sometimes loss of data due to equipment failure. To address these challenges, active research is being conducted in the field of agricultural digitalization [6,7,8], which involves the integration of modern digital technologies into the agricultural sector to enhance production efficiency, management, and sustainable development. This process encompasses a wide range of aspects, including automation [9,10,11], data-driven decision-making [12,13,14,15], water resource monitoring and management [16,17,18,19,20], and the application of innovative technologies to improve productivity [21,22,23,24,25]. The analysis of existing studies indicates that the digitalization of agriculture primarily focuses on several interrelated technological directions. One key area is precision agriculture, which relies on data collection and analysis to optimize the use of water resources. This includes the use of GPS and Geographic Information Systems (GIS) for detailed field mapping, along with drones and satellite imagery for continuous crop monitoring. Another important aspect is the Internet of Things (IoT), which enables the integration of diverse sensors to collect real-time data on water levels and related environmental parameters. In addition, big data and advanced analytics facilitate more accurate and data-driven decision-making by aggregating information such as weather patterns, crop yields, and resource use efficiency.

The specific aim of this study is to design and evaluate a low-cost, real-time water resource monitoring system based on IoT technologies. The main advantage of the proposed system lies in its affordability, the absence of the need for specialized configuration or calibration during deployment, and its capacity to provide continuous monitoring with fast access to data. Furthermore, the system supports intuitive data visualization through the use of open-source libraries, enhancing accessibility and transparency for end-users.

## 2. Related Works

In [26], the authors present a low-cost method for measuring water level in open channels using a JSN-SR04T ultrasonic sensor housed in a vertical pipe to isolate disturbances like waves, wind, and floating debris. The design uses an ATmega328 microcontroller for data processing and real-time logging on an SD card. The pipe serves as a stilling well alternative, making the water surface inside flat and calm, which increases measurement accuracy. However, the system is standalone and does not support wireless data transmission or integration into a broader monitoring network. Compared to our work, this study offers a useful solution for minimizing external disturbances in open-channel measurements. However, it lacks modern IoT architecture, data visualization, cloud storage, and a multi-layered system design. Our system overcomes these limitations by incorporating real-time wireless communication, local/cloud data storage, modular architecture, and advanced data processing. Thus, while their method simplifies physical sensing, our work provides a scalable, intelligent, and integrated solution for automated water resource monitoring. In [27], the authors developed a low-cost IoT-based system for the continuous monitoring of water levels. The system integrates sensors and a microcontroller to collect water-level data, which is transmitted to the cloud and stored in Google Sheets. A stage–volume curve derived from a contour map allows the system to estimate the volume of water in the tank at any given time. The data can be accessed via a web or mobile application, enabling farmers and water managers to make informed decisions. The system is designed to be simple, affordable, and scalable, and it can also be adapted for flood warning applications. However, the system has some limitations: it does not include predictive analytics or machine learning for forecasting water availability, lacks solar power support for autonomous operation in remote areas, and does not provide long-term field validation or integration with broader water resource management platforms. The paper [28] provides a systematic review of IoT water-quality monitoring systems, covering 50 papers published between 2018 and 2022. The review examines conceptual developments and implements solutions that use GSM, Wi-Fi, and WSN to collect data on *pH*, temperature, turbidity, and other parameters. Particular attention is given to cloud data transmission, analytics, and real-time visualization. The paper highlights key issues, such as the lack of standardization, limited sensor accuracy, and poor integration of analytical platforms. Ultimately, the authors stress the necessity of additional research to enhance the dependability and effectiveness of IoT monitoring of water resources. In [29] the authors evaluate a low-cost non-contact ultrasonic sensor for measuring water stage in an urban stream, comparing it to a pressure transducer over nearly one year. The results show stage differences within 7%, with the ultrasonic sensor readings being slightly influenced by diurnal air temperature changes, which did not affect the pressure sensor. While the ultrasonic device is practical for harsh environments where submerged instruments might fail, it remains a standalone sensor without IoT integration. The authors in [30] present a comprehensive bibliometric and systematic review of IoT applications in agricultural irrigation systems. This highlights key technologies such as low-power communication protocols, common microcontrollers, and sensor types that are widely adopted in precision agriculture. The review underscores the main challenges, including a lack of universal IoT standards, security/privacy concerns, and infrastructure limitations in rural areas. Compared to our work, their study provides a broad landscape of smart irrigation trends and technology adoption but offers no practical implementation of water-level monitoring or data processing pipelines. According to [31], the authors present an algorithm for processing water-level data collected by ultrasonic sensors in streaming conditions. The algorithm includes the stages of removing noise and smoothing. They focus on improving the quality of ultrasonic sensor data by removing noise and smoothing, while our work is aimed at developing a full-fledged IoT water-level monitoring system with a multi-level architecture, including data collection, storage, and visualization. Unlike them, we emphasize network technologies, as well as cloud and local solutions for the reliable transmission and storage of information. In [32], the authors describe the deployment of a uniform real-time network of 18 automated telemetric stations across 11 rivers in Greece, measuring *pH*, temperature, electrical conductivity, dissolved oxygen, and water stage. This work emphasizes quality-control procedures, such as range and variability tests, and identifies that *pH* and sensors require more frequent maintenance than stage, temperature, or conductivity sensors. The network includes an online platform for data visualization and timely dissemination to end-users but remains focused on the national/regional infrastructure level. In [33], the authors present a method for estimating river discharge using a cost-effective, non-contact Doppler radar sensor. This sensor is installed on a bridge or pole above the river to measure surface flow velocity. The paper validates this method against conventional current meters and demonstrates a high level of correlation, showing that low-cost radar solutions can be a viable option for continuous hydrological monitoring. The system also uses a depth–velocity rating curve to estimate total discharge; however, it does not incorporate IoT based transmission or real-time data analytics layer. In the study in [34], the authors designed and validated an innovative out-of-water radar sensor capable of simultaneously measuring water surface velocity and depth. The sensor is notable for its low cost, under USD 50, low power consumption, and non-contact design, making it suitable for high-resolution hydrological monitoring in urban and stormwater systems. It features a 3D-printed radar lens that enhances sensitivity by 21 dB and is built using open-source hardware and software. Laboratory and field tests demonstrated a depth accuracy within ±6% and velocity uncertainty ranging from <30% to 36%, making it a competitive alternative to more expensive commercial sensors. However, the system has some limitations: it does not include cloud-based data integration, real-time wireless communication, or predictive analytics capabilities. Additionally, while the sensor is validated in controlled and field conditions, its long-term durability and performance in extreme environmental conditions remain to be fully assessed. In [35], the authors designed a real-time water-level monitoring system using the NodeMCU microcontroller, an JSN-SR04T ultrasonic sensor, and a cloud-based interface via ThingSpeak. The system continuously measures the water level in a tank or reservoir and uploads the data to the cloud, where it can be visualized and analyzed remotely. The project emphasizes its low cost, ease of implementation, and suitability for domestic and agricultural water management. It also includes a mobile notification feature to alert users when water levels cross predefined thresholds. However, the system lacks advanced features such as predictive analytics, integration with broader water management platforms, or support for solar power, which limits its scalability and autonomy in remote or large-scale irrigation networks. Additionally, the paper does not provide long-term field validation or a comparative analysis with other existing systems. In [36], the authors proposes an innovative radar system that combines FMCW (Frequency-Modulated Continuous Wave) and Doppler radar modes using a frequency-scanning microstrip antenna. This dual-mode approach allows the radar to simultaneously measure surface velocity and reconstruct the river’s cross-sectional topography from a fixed station. The system provides high spatial and temporal resolution, making it effective for flood monitoring and hydrological modeling. Data is acquired and processed locally, but there is no mention of real-time transmission or modular IoT design. In [37], the authors investigated the feasibility of using low-cost ultrasonic sensors combined with a microcontroller to monitor water-level variations in open channels under laboratory conditions. The study focused on short-duration events and evaluated the accuracy of the sensors using an Acoustic Doppler Velocimeter (ADV) as a reference. The system demonstrated promising results in detecting water-level changes, offering a cost-effective alternative for hydrological monitoring in controlled environments. However, the study has several limitations: it lacks temperature compensation in measurements, which may affect accuracy; it does not include real-time wireless data transmission or cloud integration; and it was conducted only under laboratory conditions, without field validation. Additionally, the work is more technical in nature and lacks broader scientific analysis or integration with predictive or decision-support systems. In [38], the advantages of using unmanned aerial vehicles (UAVs) for analyzing the state of water resources are discussed, including the potential of drones integrated with artificial intelligence for remote water monitoring and for collecting and processing big data during research. In [39], an analysis is provided of the practical experience of using big data in agriculture, along with an overview of IT companies developing solutions based on big data technologies. Among the available big data analysis methods, the most in-demand in agriculture are data mining, machine learning, pattern recognition, spatial analysis, and time series analysis. In [40], the potential of using big data and IoT technologies for effective water resource management is explored. A comparative analysis is conducted on the use of these technologies for developing monitoring and control system architectures and algorithms aimed at addressing water scarcity. The article discusses the characteristics of the proposed architectures and outlines prospects for their development. In [41], a review of scientific articles is presented, focusing on the effective use of big data analytics and the Internet of Things in water resource management. The authors also propose a new architecture for conducting big data analytics and discuss the impact of these technologies on water management strategies. In [42], an integrated system based on IoT technologies for monitoring and managing water resources is proposed. The system consists of three main levels: the data collection level, the data transmission level, and the data application level. At the data collection level, a sensor network is created to monitor water resource information. At the data transmission level, real-time data transfer is carried out. At the data application level, functions for storing, managing, applying, and disseminating water-related information to users are implemented. In [43], the authors present examples of using big data and IoT technologies to address issues related to water loss accounting, pollution, and leak detection. Based on the results obtained, they propose a big data and IoT architecture designed for intelligent water resource management.

The novelty of our work lies in the development of a low-cost, solar-powered, real-time water-level monitoring system that uses open-source technologies and is field-tested in an arid agricultural region. Unlike many existing systems, our solution requires no complex calibration, is easily deployable, and provides continuous data access via cloud platforms, making it highly suitable for resource-limited rural areas.

## 3. Methods

Modern information technologies and systems offer powerful tools to address the challenges mentioned above. They enable scalable data processing from multiple sources, including sensors, weather stations, and satellite imagery, which helps form a comprehensive understanding of the water balance. In addition, predictive analytics allows for the forecasting of water usage and demand by analyzing historical patterns and considering climate change trends. These technologies also support the development of water-use optimization models that enhance the efficiency of resource allocation across agricultural fields according to their specific needs and the availability of water. Furthermore, monitoring systems can be implemented to track water consumption in real time and make timely adjustments based on the most current data.

Despite the technological capabilities mentioned above, implementing big data-based systems for water resource monitoring and management presents several challenges. One major issue is the lack of data standardization, as water-related data often originate from diverse sources and formats, and significant effort is required to ensure their harmonization and integration. Another challenge lies in technical limitations, particularly the need for high-performance computing resources to handle large data volumes, which can be difficult to access in remote or rural areas with limited infrastructure. Additionally, the availability of accurate and up-to-date data on water consumption and the condition of water bodies is often limited. Finally, economic and social barriers, such as the high cost of system implementation, can significantly hinder adoption, especially among small and medium-sized farmers.

To address the challenges of agricultural water management, we employed a methodology that tightly integrates Internet of Things sensing with cloud-based big data processing. We built a field-deployable sensor node powered by a small solar panel and battery to ensure 24/7 operation in remote locations. The node’s core is an ESP8266 microcontroller with Wi-Fi, chosen for its low power draw and full TCP/IP stack support. Connected to the microcontroller are an ultrasonic water-level sensor and auxiliary sensors for air temperature and humidity. All sensor signals are read and processed by the ESP8266, which we programmed using the Arduino IDE and relevant libraries for sensor interfacing. The firmware implements data acquisition at regular intervals, basic filtering, and the wireless transmission of readings. Notably, our design requires no complex on-site calibration beyond an initial installation setup. In our method, each sensor node communicates over the wireless network to transmit data in real time to a cloud server. Sensor readings are sent via lightweight HTTP requests to a cloud endpoint, which then inserts the data into a cloud-hosted PostgreSQL database. This design ensures that raw data is immediately centralized for analysis and avoids the need for local data logging. By using existing internet infrastructure, we keep costs low and achieve virtually unlimited spatial access to the data. The choice of a relational database was driven by its support for large datasets, time-series queries, and spatial extensions, aligning with big data best practices in water resource management. We also set up Google Cloud Storage as a data lake for unstructured or large data. This methodological step connects our IoT device layer with scalable cloud services, embodying IoT and big data. With the data streaming into the cloud, we implemented user interface components to close the loop for end-users. Our methodology includes a data processing layer where incoming sensor data is analyzed for trends, anomalies, and predictions. For the user interface, we built a web-based dashboard that visualizes the canal’s water level in real time, along with temperature, humidity, and battery status. The complete system was field-tested at the Left Bypass Canal (LBC) in Taraz, Kazakhstan under real agricultural conditions. The collected dataset was analyzed to evaluate measurement accuracy and system performance. Importantly, the system proved effective for capturing dynamic changes, that would be hard to monitor manually. Through these methods, we established a robust and scalable approach for real-time water-level monitoring in agriculture, uniquely characterized by its low cost, energy autonomy, and integration of big data techniques for resource management.

## 4. System Architecture

### 4.1. System Requirements

To design an effective system architecture, it is essential to first define the key system requirements in a precise and comprehensive manner. This foundational step ensures that the proposed solution not only addresses both current and anticipated user needs but also lays a solid groundwork for scalable, adaptable, and sustainable implementation in diverse operational environments. To achieve this objective, several essential system requirements have been identified and elaborated. First and foremost, the system should provide a comprehensive set of core functionalities required for robust and efficient water resource monitoring. These include remote data acquisition capabilities for collecting critical information on the state of water bodies, such as flow rate, water level, and temperature, along with operational data from physical devices, including system health status and battery levels. In addition, the system must support remote control of actuators and centralized storage of the collected data in a secure and specialized database, offering uninterrupted 24/7 access and intelligent data processing capabilities for real-time and historical analysis. Moreover, the system must be capable of seamless interaction with external applications and systems to support data exchange related to environmental variables, such as soil conditions, weather patterns, and other relevant ecosystem factors. To ensure compatibility across a wide range of deployments, a flexible and scalable system architecture is crucial. This flexibility guarantees interoperability between hardware and software components, while also supporting the integration of heterogeneous technologies and devices across different layers of the system. To enable smooth communication between architectural components, the system must incorporate open-type interfaces and adopt relevant IoT standards and protocols, particularly those applicable to water management applications. These interfaces allow for standardized data exchange and integration with third-party solutions or emerging technologies in the sector. Finally, the architecture must be fully compatible with the existing infrastructure and equipment already installed at water management facilities. This includes the ability to integrate with subsystems operating as part of a unified infrastructure, which may rely on embedded communication modules and protocols tailored to specific environmental and operational conditions.

**Figure 1:**
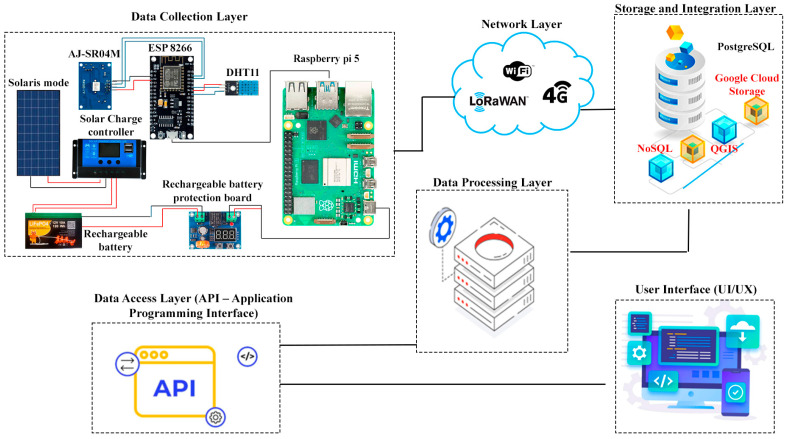
System architecture.

As illustrated in Figure 1, based on the defined set of requirements, we developed a six-layer architecture tailored for real-time water-level monitoring. This section provides detailed technical descriptions of each architectural layer, outlining the specific technologies, hardware components, communication protocols, and design rationale that guided our implementation. The layered structure is purposefully designed to enhance modularity and system scalability, enabling each layer to evolve independently based on technological advancements or functional demands. This separation of concerns reflects best practices in contemporary IoT reference architectures and ensures that the proposed solution remains compatible with other platforms while accommodating future extensions or enhancements.

### 4.2. Data Collection Layer

**Figure 2:**
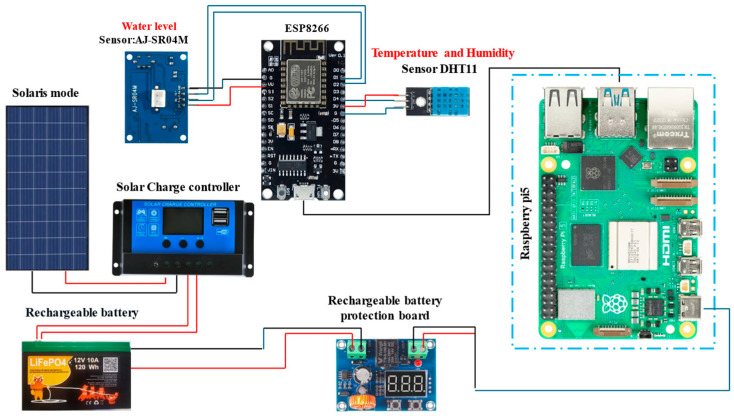
Architecture of the data collection layer.

Figure 2 shows the data collection layer, designed to gather data and other environmental information necessary for monitoring and decision-making regarding water resources. It was implemented using industrial microcontrollers, renewable energy sources, and cloud technologies, enabling it to function effectively in remote or autonomous environments. The data collection layer includes the following components: (1) The solar panel converts solar radiation into electricity and supplies it to a battery to ensure uninterrupted power for the monitoring system. Due to the high solar activity in the region and the system’s low energy consumption, even a small solar panel is sufficient for this function; (2) A battery with a power converter is designed to accumulate and store electrical energy from the solar panel and supply power to all system components, including the microcontroller and sensors. A voltage stabilizer allows for the continuous operation of electronic devices for round-the-clock monitoring of water levels and environmental conditions; (3) The system includes a solar charge controller, which plays a crucial role in managing the power supply when using solar energy as the primary source. This component regulates the voltage and current coming from the solar panels to the battery, ensuring efficient charging while preventing overcharging, deep discharging, and power fluctuations. By maintaining battery health and stability, the solar charge controller contributes to the autonomous and sustainable operation of the system, particularly in remote areas where a reliable electrical grid may not be available. (4) Microcontroller ESP8266 (Espressif Systems, Shanghai, China) serves as the central control unit. According to the program stored in its memory, it processes data from connected sensors (temperature, humidity, and water level), establishes a wireless connection with cloud storage, and transmits the data. The ESP8266 board supports Wi-Fi connectivity with full TCP/IP stack capabilities based on a standalone microcontroller. This module allows microcontrollers to connect to Wi-Fi networks and establish simple TCP/IP connections using AT commands. The low cost and minimal number of external components on the module—and, consequently, the compactness and cost-efficiency of the system based on it—make this component the most suitable for our purposes; (5) Air temperature and humidity sensor DHT11 (ASAIR, Guangzhou, China) measures ambient air temperature and humidity. It monitors climatic conditions that may affect water evaporation rates and other environmental parameters. The data recorded by this sensor can be analyzed to generate predictive assessments of water resource conditions and support informed decision-making; (6) The water-level sensor, based on JSN-SR04T ultrasonic distance module, provides non-contact real-time measurements of the current water level in a reservoir. The module is housed in a durable waterproof casing and includes an integrated ultrasonic transceiver operating at an acoustic frequency of 40 kHz, along with a control circuit. It features low power consumption, with an operating voltage of 5V and current 30 of mA, and functions in the temperature range from −10 °C to +70 °C; (7) Raspberry Pi 5 serves as the processing unit for the system, while the Arduino IDE development environment is utilized for writing, debugging, and uploading the C++ code to the ESP8266 microcontroller. The Arduino IDE is a user-friendly and versatile platform that supports a wide range of development boards and libraries, making it well-suited for microcontroller programming in this application. Sensor data, including water level, temperature, humidity, and corresponding timestamps, is periodically collected and stored in a structured database. The Raspberry Pi 5 is equipped with its own local data storage, which allows for the temporary retention and processing of collected information. However, due to its limited storage capacity, a dedicated Storage and Integration Layer was incorporated into the system architecture. This layer is responsible for managing, aggregating, and processing data before transferring it to long-term storage or cloud-based services.

### 4.3. Network Layer

Recently, many context-aware systems have been developed using embedded sensors. Additionally, numerous studies utilize Wireless Sensor Networks composed of single-board microcomputers equipped with sensors to more accurately determine environmental conditions [44,45]. As shown in Figure 1, the network layer is responsible for data transmission and connectivity between field devices and the cloud infrastructure. In our implementation, the network layer is built on a wireless communication link using Wi-Fi. The ESP8266 built-in Wi-Fi module connects to an internet gateway; in our case, this is a 4G LTE router deployed at the canal site to send data to the cloud. The choice of Wi-Fi was motivated by its ubiquity and sufficient range in the test environment; however, the architecture is modular, allowing alternative long-range technologies like GSM/GPRS to be used in areas without Wi-Fi coverage. The network layer comprises two sub-components. In the context of the system under consideration, the access network is defined as the local Wi-Fi link between the sensor node and the router. The transport network, however, is defined as the internet path from the router to the cloud server. The transmission of data from the sensor node is achieved through the utilization of HTTP POST requests over TCP/IP, a method that can be considered as straightforward given the fact that the ESP8266 is equipped with a complete TCP/IP stack. The implementation of a basic RESTful endpoint on the cloud, utilizing a Python version 3.12, was undertaken for the purpose of securely receiving the aforementioned incoming data packets. The REST API utilizes the HTTP protocol over the standard IP network, thereby providing a uniform and language-agnostic method for data upload. The decision to utilize REST/HTTP was made on the basis of its compatibility with the microcontroller and the ease with which it could be debugged. However, should the necessity arise for more lightweight, publish/subscribe communication, the system could be extended to utilize Message Queuing Telemetry Transport. The interoperability of the network layer is of significance, and the design adheres to IoT standards so that additional sensor nodes or gateways can be integrated without significant changes. In summary, the network layer ensures that sensor readings are seamlessly and rapidly conveyed from the field to the cloud, leveraging the existing communication infrastructure for cost efficiency.

### 4.4. Storage and Integration Layer

The layer includes components that ensure secure data storage, optimize access and manage metadata. Once data reaches the cloud, it enters the storage and integration layer. This layer handles secure data storage, organization, and integration with other data sources. At its core is a Cloud Database service where all incoming measurements are archived. We utilized PostgreSQL as the primary Database Management System for structured sensor data. PostgreSQL was chosen for its reliability, ACID compliance, and support for advanced data types, which could be useful for the geospatial tagging of sensor locations. In addition, PostgreSQL’s open-source nature and scalability align with our goal of a low-cost yet robust system. To ensure reliable storage of the collected data, we use local university servers in parallel with Google Cloud Storage. This allows for the storage of unstructured or large-scale data that may be integrated in the future, for example, UAV imagery of crop fields or longer time-series logs exported for machine learning. The data lake concept ensures that all data, regardless of format, can be retained and later transformed for analysis. The implementation of an ETL (Extract, Transform, Load) process was undertaken in order to facilitate the movement of data from its raw ingestion state to a usable form. The implementation of a dual-tier storage system, encompassing both raw and processed data, has been demonstrated to enhance reliability and facilitate flexibility in data processing operations. Furthermore, the integration layer is configured to combine our sensor data with external streams, such as weather API data or soil moisture readings from other projects, by cross-referencing timestamps and location identifiers. The database schema was designed with the intention of accommodating such integration, following standard water data ontologies wherever possible in order to facilitate interoperability. In order to optimize query performance on the growing dataset, indexing by time and sensor was added, and PostgreSQL’s native JSON/B support for semi-structured data was utilized. Finally, this layer ensures data governance. It is imperative to note that user access controls, backup routines and data retention policies are enforced at the database level in order to ensure the security of the information. By centralizing all collected data in a structured, scalable storage layer, the foundation is established for advanced analytics and cross-domain insights into water usage.

### 4.5. Data Processing Layer

At this layer, the system performs an analysis of current environmental parameters, such as air and water temperature, soil moisture, and others, along with forecasting water consumption and precipitation levels and optimizing water resource management. The components of this layer are used to interconnect various platforms within IoT sensor environments. Key components of this layer include real-time stream processing for immediate calculations and batch processing for a deeper analysis of historical data. For real-time processing, each incoming water-level reading is passed through a moving-average filter to smooth out momentary spikes or sensor noise. We also apply outlier detection rules. If a reading deviates beyond a reasonable range or changes more rapidly than physically possible based on canal flow dynamics, it is flagged for review. The filtered data is then used to compute summary statistics and is stored in separate analytics tables, allowing the dashboard to query the data efficiently. In parallel, batch processing supports more complex analysis. We utilize cloud functions to run scheduled tasks, such as daily analyses of water-level patterns and their correlation with weather conditions. Over longer periods, the accumulated data is used to train predictive models. These models may begin with simple regression or time-series approaches. The goal is to forecast short-term changes in water levels or demand, supporting proactive decision-making in water allocation. This data processing layer also handles alert generation. If certain thresholds are crossed, such as critically low or high-water levels, the system generates an event that is transmitted to the UI layer or sent via notifications. Importantly, this layer is built on the cloud infrastructure, which allows it to scale as needed. As the volume of data increases, computing power can be expanded or distributed processing can be implemented.

The purpose of this design is to ensure that the system not only monitors data but also interprets it and provides intelligent feedback. By using cloud computing, the limitations of edge device processing are addressed, in line with best practices in IoT-based resource optimization. This data processing layer effectively transforms the system from a simple data logger into a decision-support tool capable of analyzing water trends, forecasting needs, and detecting inefficiencies such as unusual losses or leaks in the irrigation network.

### 4.6. Data Access Layer (API—Application Programming Interface)

The API layer provides access to data and analytics for external systems, mobile applications, and web platforms. The implementation of this layer was undertaken as a RESTful Application Programming Interface (API), in accordance with the Representational State Transfer design principles, in order to ensure simplicity and compatibility. A set of REST endpoints was developed to expose various functionalities. Furthermore, we contemplated the integration of more specialized interfaces for high-frequency data access or the dissemination of updates to clients. The potential addition of a WebSocket or Server-Sent Events channel would facilitate the real-time streaming of data to dashboards. The API employs standard HTTP methods, namely GET for retrieval, POST for the transmission of new data or commands, and so forth. The data is formatted in JSON for lightweight interchange. Internally, the API interacts with the storage layer via SQL queries using a server-side library with connection pooling to the PostgreSQL database and with the processing layer by invoking functions or retrieving pre-computed results. It was imperative that the API be documented using an OpenAPI/Swagger specification, thus facilitating seamless integration with other developers’ systems; for example, a mobile application designed for farmers. The communication protocols supported by the API layer include HTTP/HTTPS for web clients and MQTT for IoT interoperability. To illustrate this, data can be published to an MQTT broker to which other subscribers can listen. By providing this data access layer, we ensure that valuable information collected and processed by the system is accessible to end-users in real time and can be integrated into larger agricultural management systems or decision support tools. This creates an open interface between our water monitoring system and external applications, facilitating data sharing and increasing the usefulness of the system beyond its own user interface.

### 4.7. User Interface (UI/UX)

The top layer of the architecture is the user interface, which delivers the visualized data and interactive functionality to end-users. The UI layer was implemented as a web application, which can be accessed via standard web browsers on desktops, tablets, or smartphones. The decision was taken to adopt a web-based approach with the objective of optimizing accessibility and facilitating rapid iteration on the interface. The UI provides dashboards displaying the current water level of the canal, historical trends, and environmental conditions. For instance, the main dashboard view presents a time-series graph of water level alongside plots of temperature and humidity over time. This facilitates the correlation of meteorological conditions with the availability of water resources. The front-end was constructed utilizing a contemporary JavaScript framework in conjunction with visualization libraries to facilitate the generation of interactive graphs. We have also implemented features such as map integration to display sensor locations on a map of the canal network. Given that field personnel might check data on a smartphone, the UI is responsive and mobile-friendly. We used a CSS framework to ensure a consistent and adaptive layout. Although our current deployment is for monitoring only, we designed the UI with future expansion in mind and reserved space for control dashboards. In terms of the backend, we deployed the UI on a cloud web server with an auto-refresh mechanism so that data updates without a full page reload. We also considered creating a native mobile app for offline scenarios, but a well-optimized web app serves the purpose for now. Our UI layer design is intended to make the complex data generated by the system easily understandable and actionable. By presenting processed information such as fill percentage or trend predictions in a user-friendly format, the UI enables stakeholders to leverage the system for better decision-making. Ultimately, this layer closes the feedback loop from data collection to user action, thus embodying the system’s purpose of enabling informed, real-time water management in agriculture.

### 4.8. Water-Level Calculation Algorithm and Testing

The calculating algorithm of the water-level monitoring system is based on measurements of the time it takes for the ultrasonic signal to travel from the sensor to the water surface and back. The obtained distance data is converted into water-level data by considering the height at which the sensor is installed above the maximum water-level mark. The system is connected to an ESP8266 microcontroller, which processes the signal, filters out noise, and converts the data into digital format. The ultrasonic sensor operates using the trigger and echo method. The transceiver module emits a signal toward the water surface, and the reflected echo signal is received by the receiver module. The ultrasonic sensor calculates the distance and returns the value of the distance to the water surface. The time of flight and the speed of sound are used by the sensor to calculate the water level. The ultrasonic wave is emitted by the transmitter through the Trigger pin, which receives a pulse signal from the microcontroller. The reflected signal is then received by the receiver and sent to the microcontroller via the Echo pin. The ultrasonic wave is emitted when the sensor receives a 10 μs pulse signal through the Trigger pin. The emitted and reflected ultrasonic wave has a frequency of 40 kHz. The measured time delay between transmission and reception is used as a reference to calculate the distance from the sensor to the water surface. The distance calculation formula is as follows:

(1) $$d=\frac{t\times v}{2}$$

where *d* is the distance to the water surface (in meters); *t* is the time of signal return (in seconds); *v* is the speed of sound in air (approximately 343 m/s at an air temperature of 20 °C).

The speed of sound depends on the air temperature above the water. For example, at 30 °C, the speed of sound increases to approximately 349 m/s, which requires adjustment in the calculation. Measurement accuracy is also affected by air humidity and condensation: high humidity or condensation on the sensor (despite its waterproof design) can reduce the accuracy of the readings. The software module, implemented on the Arduino platform, measures distance to the water surface using the AJ-SR04M ultrasonic sensor, followed by the calculation of the water level and percentage of reservoir or canal fill. The key stages of data processing and the formulas used in the program are presented below:

1. Measuring the Distance to the Water Surface: First, the built-in *pulseIn()* function is used to measure the time it takes for the ultrasonic signal to travel from the sensor to the water surface and back. Based on this time, the distance is calculated using the following formula:
  *distance = (duration × SPEED_OF_SOUND)/2* (2)
  where duration is the measured time in microseconds, i.e., the time it takes for the ultrasonic pulse to travel to the water surface and back (in microseconds); SPEED_OF_SOUND is the speed of sound in air (approximately 0.0343 cm/µs); distance is the distance from the sensor to the water surface, in centimeters. Division by 2 is used because the measured time includes both the forward and return path of the signal.
2. Calculating the Water Level: Since the sensor is installed at a specific height above the bottom or baseline (SENSOR_HEIGHT), the current water level is calculated as follows:
  *water level = MAX_DISTANCE − (distance − SENSOR_HEIGHT)* (3)
  where *MAX_DISTANCE* is the maximum expected water level in the system (e.g., the height of the reservoir); *SENSOR_HEIGHT* is the height at which the sensor is mounted above the zero-reference point. This allows the system to determine the actual height of the water column relative to the bottom.
3. Calculating the Relative Water Level (as a Percentage): For easier visualization and monitoring, the water level is also expressed as a percentage of the maximum allowable value:
  *water percentage = (water level/MAX_DISTANCE) × 100.0* (4)

This value can be used for level indication in control systems, visual interfaces, or alerts for critical changes. The proposed software algorithm provides an automatic and continuous assessment of the water level with high accuracy. It is easily adaptable to various operating conditions and can be integrated into broader remote monitoring systems. To ensure reliable monitoring, the system includes periodic calibration, as well as built-in functions for value-averaging, outlier elimination, and anomaly detection. The collected data can be transmitted to a remote server via wireless communication modules, enabling real-time remote monitoring of water levels.

**Figure 3:**
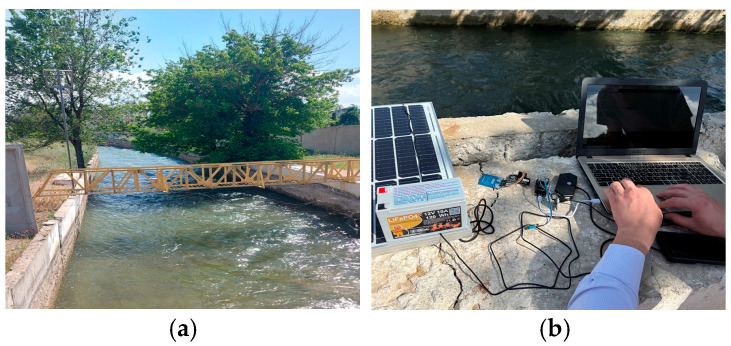
Test sites: (**a**) hydrological post of the left bypass canal; (**b**) field testing of the water level monitoring system model.

Testing of the system model was conducted at the LBC in the city of Taraz, located in the Zhambyl Region, as shown in Figure 3a,b. The LBC is managed by the Republican State Enterprise “Kazvodkhoz”, which is a critical hydraulic structure that plays a key role in supplying water to the region’s agriculture, especially in the arid zones of the Zhambyl Region. Surface velocity measurements were carried out on the bridge at a distance of 3 m from the canal surface.

The field test involved the continuous collection of data over a period of several weeks, resulting in a total of 703 h of data being recorded. The environmental conditions during the experimental phase included elevated daytime temperatures (up to 39 °C) and nocturnal temperatures (approximately 23 °C). The system transmitted data in real time via the 4G router to the cloud. The incoming data was monitored through the utilization of a dashboard, and an automated log was also configured.

**Figure 4:**
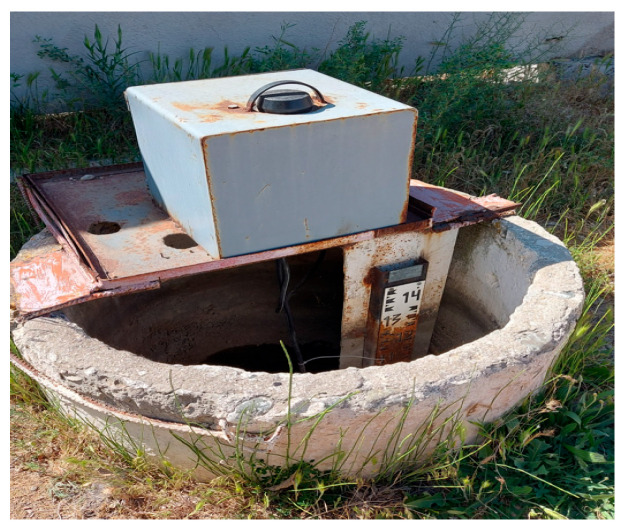
Staff gauge of the LBC.

As illustrated in Figure 4, the water level in the canal is measured using a staff gauge installed in a special concrete reservoir. While the staff gauge is simple and reliable, it presents several significant drawbacks, especially in situations where timely and accurate water resource management is required. For example, reading the gauge requires a person to be physically present at the installation site. This is particularly inconvenient during bad weather, during floods, or in remote areas far from populated locations. The staff gauge is not connected to digital systems, so data cannot be transmitted in real time. This limits its use in big data systems, automated control, and forecasting. The absence of lighting or a digital display makes nighttime or emergency measurements difficult. In winter, the staff gauge can freeze over or be buried under snow, rendering it unusable. During floods or strong currents, the scale may be damaged or submerged.

Human error is possible when reading values, especially if the scale is dirty, worn, or difficult to see. Readings must be recorded manually, which increases the risk of data loss and makes trend analysis inconvenient. Without automation, it is impossible to assess long-term water-level dynamics. Thus, under modern conditions, the staff gauge no longer meets the requirements for accurate, timely, and automated water management—especially in agricultural regions with high irrigation demands. The developed model ensures real-time responsiveness and accuracy, which is especially important in the context of the digital transformation of the agro-industrial complex.

## 5. Results

**Figure 5:**
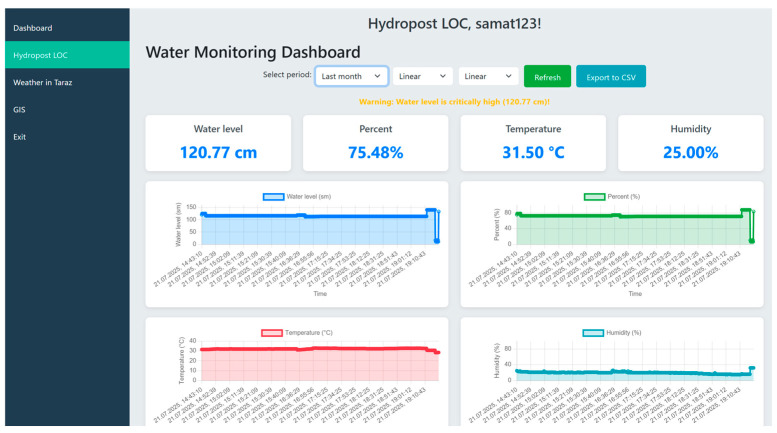
Real-time level readings.

Figure 5 illustrates the water monitoring parameters at the observation post, showing data such as water level, filling percentage, air temperature, relative humidity, date and time.

**Figure 6:**
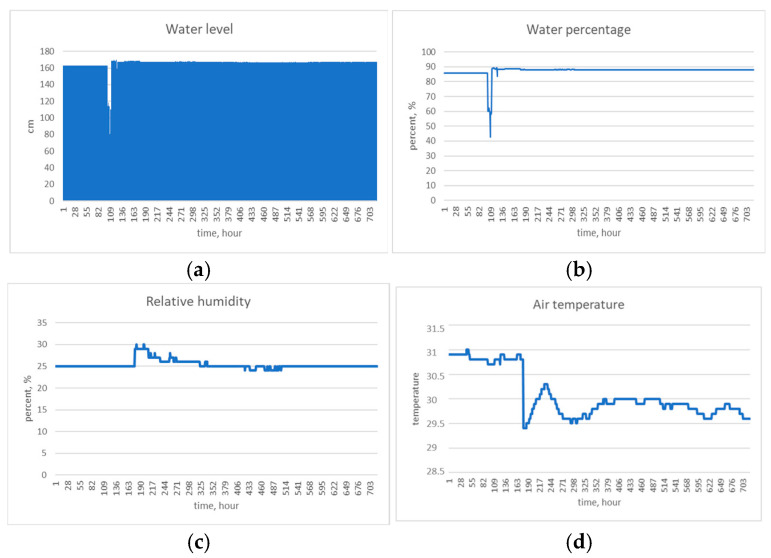
Monitoring results for 703 h: (**a**) water level; (**b**) water percentage; (**c**) relative humidity; (**d**) air temperature.

As shown in Figure 6, the data were collected hourly and stored in a database. These data can be used for analysis and decision-making in agriculture, water resource management, and the prevention of hydrological risks. For example, analyzing the dynamics of water levels makes it possible to identify patterns that indicate the risk of floods or droughts. Data on relative humidity and air temperature are useful for assessing evaporation and the water balance, which is important for forecasting dry periods. Most importantly, data on water percentage and level help plan irrigation cycles, avoiding water overuse or shortages.

As the canal was actively managed for irrigation supply during this period, the water level varied between about 160 cm and 170 cm, as shown in Figure 6a. The ultrasonic sensor closely tracked these variations, capturing gradual changes and sudden drops; for example, when water was released downstream. The readings showed minor diurnal fluctuations of ±1–2 cm, even on days when irrigation was static. This can be attributed to the temperature-induced expansion of water and possibly minor sensor drift, but it remains within acceptable error limits. As illustrated in Figure 6b, the water percentage mostly remained between 80% and 90% of the canal’s capacity. This information is directly useful to water managers when deciding whether to divert more water into the canal. Figure 6c,d show the measurements of relative humidity and air temperature recorded by the DHT11 sensors. As expected, an inverse correlation was observed between dry, hot afternoons and cooler, humid nights. More importantly, the temperature data was used in our compensation formula for the speed of sound, improving accuracy on hot days. On one particularly windy afternoon, we observed that the water surface was rough and the ultrasonic readings had slightly higher noise, around ±1 cm variance. This was temporary, however, and the filtering in the cloud removed most of that noise from the final dataset.

The system’s performance was found to be satisfactory, in accordance with the design specifications. The precision of field-based water-level measurements was found to be within the range of 1–2 cm. The real-time aspect was demonstrated to be fully functional, with data being made available on the dashboard within a time frame of 2–3 s after measurement. This low latency is imperative for any future integration with control systems, for instance, automatically adjustments to a gate if the water level is too low. The system also demonstrated reliability, with no system crashes or data loss observed during the 703 h continuous run. The Wi-Fi/4G network link exhibited consistent stability, and the solar panel and battery ensured uninterrupted power supply. Despite a few days of cloud cover, the power system maintained sufficient reserve capacity. The findings of this study suggest that a cost-effective IoT strategy can be successfully employed to ensure the reliability required for critical water monitoring applications. From a water management perspective, the insights provided by our test deployment are of value. To illustrate this point, an analysis of the data revealed instances of elevated water levels that were not necessary for the present irrigation requirements. This finding indicates potential for reducing inflow and conserving water. These findings serve to substantiate the system’s capacity to enhance water-use efficiency and curtail non-productive losses.

The developed monitoring system model demonstrates high resistance to external influences, minimal energy consumption, and ease of installation, making it suitable for autonomous operation in field conditions. This approach enables prompt responses to flood events and other hydrological risks, improving the accuracy and frequency of observations compared to traditional methods. Thus, the use of this system in agriculture offers several key advantages. The first is autonomy and energy independence. The use of a solar panel and battery allows the system to operate in remote areas without connection to the power grid, which is especially relevant for agricultural regions lacking stable electricity supply. The second is remote monitoring and control. Real-time data is transmitted to cloud storage and made accessible from any device. Integration with a mobile application is possible for notifications and further data analysis. Thirdly, the system is easy to expand; additional sensors and modules can be connected. The use of open technologies ESP8266, Arduino and PostgreSQL makes it flexible and customizable to specific needs. Fourthly, the system demonstrates environmental friendliness and resilience. Solar energy usage reduces the carbon footprint and lowers operating costs. The system’s high resistance to external factors is ensured by the use of standard industrial components, enhancing reliability through compliance with established requirements and enabling quick replacement in case of failure. Finally, the system is low-cost and accessible. The electronic components used in the system are readily available on the market at low prices, making the solution suitable even for small-scale farms with limited budgets.

## 6. Discussion

An analysis of existing approaches indicates that the implementation of monitoring systems significantly enhances water resource management processes. In particular, such systems can effectively forecast water demand across different regions, determine optimal irrigation schedules, and prevent excessive water use. However, many proposed methods and models face challenges related to data accuracy, sensor quality, and the integration of diverse information sources. Despite these issues, advancements in technologies and improvements in data collection and processing infrastructure open new opportunities for creating more efficient and sustainable water management systems in agriculture. The issue of effective water resource management in agriculture is multifaceted and requires the integration of various innovative technologies. Using statistical models or machine learning will help predict extreme events based on historical data. To better illustrate the novelty of our system, we provide a comparative analysis with the existing water monitoring solutions presented in [16,40,42], as demonstrated in Table 1. The comparison highlights key aspects, such as cost, energy autonomy, real-time capabilities, sensor integration, and ease of deployment.

Based on the developed system, our further work will be aimed at improving the system, expanding its functionality and developing a mobile application that analyzes water levels and other parameters in real time, sending notifications to farmers or authorities when critical thresholds are reached. This will significantly improve the efficiency of water use and reduce the environmental impact in the agricultural sector.

## 7. Conclusions

This study introduces a real-time water-level monitoring system that tackles the pressing challenge of water resource management in agriculture. By leveraging IoT sensors, cloud technologies, and data analytics, the system delivers accurate, timely data on water levels and environmental conditions. Its successful implementation at the Left Bypass Canal in Taraz, Kazakhstan, highlights its ability to optimize water use, minimize losses, and promote sustainable farming practices. The system’s autonomy, energy-independence via solar power, and cost-effectiveness make it ideal for remote and resource-limited areas. Future efforts will focus on enhancing functionality through machine learning for predictive analytics and developing a mobile application to provide real-time alerts to farmers and authorities, further advancing water-use efficiency and sustainability in agriculture.

## Figures and Tables

**Table 1:** Comparative analysis of IoT-based water-level monitoring systems for rivers and irrigation canals.

Feature/System	IoT-Based System	Big Data and IoT	Smart Metering	Our System
Real-time monitoring	Partial	Yes	Yes	Yes
Low-cost hardware	No	No	No	Yes
Solar-powered/autonomous	No	No	No	Partial
Open-source technologies used	No	Partial	No	Partial
Easy deployment and calibration	No	No	No	Yes
Cloud integration and remote access	Yes	Yes	Yes	Yes

## Data Availability

Data can certainly be provided upon request.
